# Supplementary material for: Resilience or robustness: identifying topological vulnerabilities in rail networks
Source: R Soc Open Sci. 2019 Feb 6;6(2):181301. doi: 10.1098/rsos.181301 (PMC6408419; doi:10.1098/rsos.181301)
Supplement: Robust data testing [file rsos181301supp1.pdf]

Supplementary Material

Main Paper: **Resilience or Robustness: Identifying Topological Vulnerabilities in Rail Networks**.
Published in **Royal Society Open Science**.
DOI: http://dx.doi.org/10.1098/rsos.181301

Alessio Pagani[1], Guillem Mosquera[1,2], Aseel Alturki[3], Samuel Johnson[5], Stephen A. Jarvis[3], Alan Wilson[1], Weisi Guo[1,4] and Liz Varga[6]

[1]The Alan Turing Institute, London, UK
[2]Mathematics Institute, [3]Department of Computer Science, and [4]School of Engineering, University of Warwick, Coventry, UK
[5]School of Mathematics, University of Birmingham, Birmingham, UK
[6]School of Management, Cranfield University, Cranfield, UK

rsos.royalsocietypublishing.org R. Soc. open sci. 000000

Alternative Data Testing

The rail performance statistics used in the main paper (Resilience or Robustness: Identifying Topological Vulnerabilities in Rail Networks) are relative to the year 2016/17, here we show this is not a spurious result by using alternative years data. We perform the same analyses using the rail statistics relative to two different years. On one hand, we compare the resilience and robustness measures with the statistics relative to the year 2012 (2011/12 Q4), the same year of the census dataset used to create the morning peak hours network. On the other hand, we compare the same measures with the rail statistics relative to the year 2018 (2018/19 Q1), the latest currently available. All the performance statistics are publicly available on the *Office of Rail and Road* website (please refer to the main document for details).

(a) Year 2011/12

In this section we discuss the correlation between the resilience and robustness measures and the PPM and CaSL relative to the year of the census used to create the morning peak hours network (2011/12 Q4).

The new results (Figure 1) confirm the ones in the previous analysis: as for 2017, we found a correlation between the normalised trophic incoherence ($q/\tilde{q}$) and the oPPM and CaSL measures (Figure 2).

(b) Year 2018/19

In this section we compare the resilience and robustness measures of the different rail networks with the latest statistics available (2018/19 Q1). We also extended the study to two more rail companies: the *Thameslink* and the *Southern Railway*. Moreover, *South West Train* has been renamed as *South Western Railway*.

The new results (Figure 3) confirm the ones in the previous analysis: as for 2017, we found a correlation between the normalised trophic incoherence ($q/\tilde{q}$) and the oPPM and CaSL measures (Figure 4).

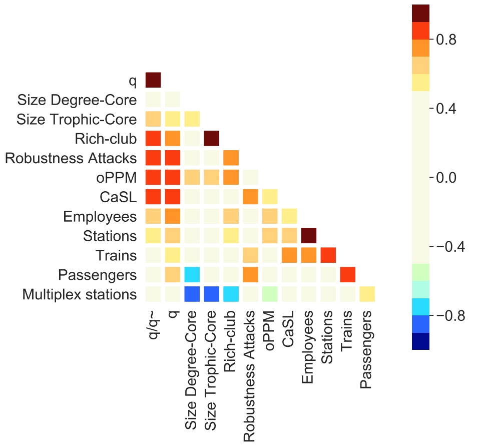

Figure 1. Year 2011/12: Pearson Correlation Coefficient between different measures and indicators (please refer to the main document for details).

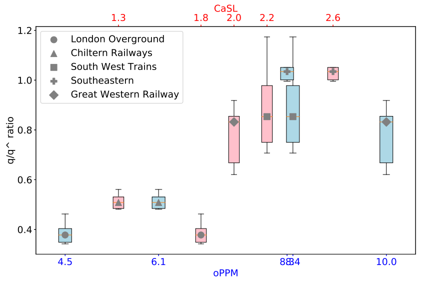

Figure 2. Year 2011/12: oPPM and CaSL compared with trophic incoherence parameter of each rail company.

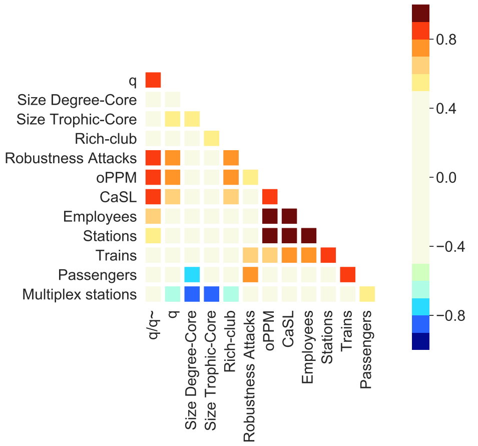

Figure 3. Year 2018/19: Pearson Correlation Coefficient between different measures and indicators (please refer to the main document for details).

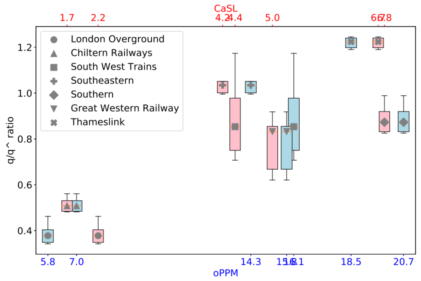

Figure 4. Year 2018/19: oPPM and CaSL compared with trophic incoherence parameter of each rail company.
