# Supplementary material for: Resilience or robustness: identifying topological vulnerabilities in rail networks
Source: R Soc Open Sci. 2019 Feb 6;6(2):181301. doi: 10.1098/rsos.181301 (PMC6408419; doi:10.1098/rsos.181301)
Supplement: Operator focused case study [file rsos181301supp2.pdf]

Supplementary Material

Main Paper: **Resilience or Robustness: Identifying Topological Vulnerabilities in Rail Networks**.
Published in **Royal Society Open Science**.
DOI: http://dx.doi.org/10.1098/rsos.181301

Alessio Pagani[1], Guillem Mosquera[1,2], Aseel Alturki[3], Samuel Johnson[5], Stephen A. Jarvis[3], Alan Wilson[1], Weisi Guo[1,4] and Liz Varga[6]

[1]The Alan Turing Institute, London, UK
[2]Mathematics Institute, [3]Department of Computer Science, and [4]School of Engineering, University of Warwick, Coventry, UK
[5]School of Mathematics, University of Birmingham, Birmingham, UK
[6]School of Management, Cranfield University, Cranfield, UK

Case study: How could adding or removing a single link improve the rail network performances?

We here propose a case study on the *Thameslink* rail network morning peak hours. Aim of this work is to show how a single modification in the rail network structure could improve the morning peak hours commuting performance. Given the correlation between the the trophic incoherence and the rail performance measures (oPPM and CaSL) demonstrated in the main paper (Resilience or Robustness: Identifying Topological Vulnerabilities in Rail Networks), we propose possible network modifications that reduce the first in order to improve the seconds.

We look for changes in the network that improve the trophic coherence without causing major disruptions in the service. In this case study, we focus on a single modification: we add and remove a link and we measure the resulting trophic incoherence, we then analyse in details a scenario with a link removed or one with a link added.

(a) Removing a link

Removing a link in the morning peak hours network can lead to major disruption (e.g., isolate parts of the network) and lengthen the commutation time. We detect these possible problems by measuring two parameters:

- Closeness: closeness measure the centrality of a node in a network and it is thus related to the commutation time.
  Closeness: $C_i = \dfrac{n-1}{\sum_j d_{i,j}}$
  We compute the average of the nodes' closeness in order to have a standard measure for comparison. We use the average closeness (even if the majority of the nodes are not affected by a single modification), because we are interested in how the whole network is affected by a single link removal (the bigger is the decrease the more the average commutation time increases).
- Largest connected component: isolation of parts of the network should be avoided, for this reason we measure the size of the largest connected component and if it decreases, the possible link removal is discarded.

We removed each node one by one, each time computing the new trophic incoherence, the average closeness and the size of the largest connected component. We then selected the link that provides the best improvements without breaking the discussed constraints.

Our analysis suggests that removing the link from *Denmark Hill* to *Elephant and Castle* provides the best incoherence gain without isolating parts of the network and without a huge increase of the commutation time. This modification improves the trophic incoherence by 41.4% and it reduces the average node closeness of around 10%.

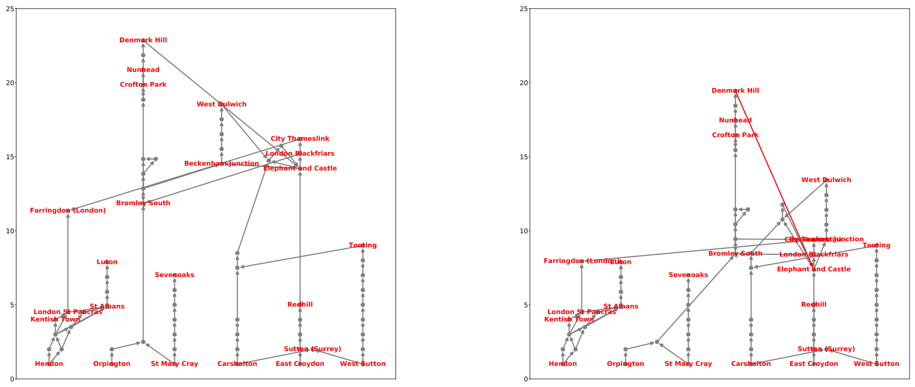

Figure 1. Stations trophic level. On the left the trophic level of the *Thameslink* stations, on the right the trophic level of the same stations when the link from *Denmark Hill* to *Elephant and Castle* is removed.

Trophic Incoherence Analysis In Figure 1 we show the incoherence gain of the *Thameslink* network when the *Denmark Hill - Elephant and Castle* link is removed. *Denmark Hill* station, the node with the highest trophic level, decreases from trophic level of 22.8 to 19.4. While this reduction influences the trophic incoherence, the main contribution in its abatement is due to another factor: loops are linked to trophic coherence and removing them improves the network trophic structure. In our case study, we detected a loop in the railway section between *Blackfriars* and *Bromley South* (see Figure 2). This loop is composed of several stations, including *Denmark Hill* and *Elephant and Castle* and deleting the proposed link (and thus breaking the loop) is the main factor that foster the trophic incoherence reduction.

(b) Adding a link

Adding a link does not cause disruptions in the network, but it might not be possible due to physical / technical constraints. We measure the impact of a new link comparing its length with the average length of the links in the network. As a rule of the thumb, we assume that if a link is too long and there are not pre-existent tracks, then the modification in the real network could be difficult or not convenient and we discard the option.

After simulating the insertion of each possible link, our analysis shows that adding a link from *Bickley* to *Nunhead* would be an optimal choice because it provides a significant decrease of the trophic incoherence. Adding this link improves the trophic incoherence of the network by 30.9%. It is longer than the link average (2.9x average length) but we believe that adding this particular link is possible since a track from *Binckley* and *Nunhead* is already available and thus it would just require to add a direct train from those two stations.

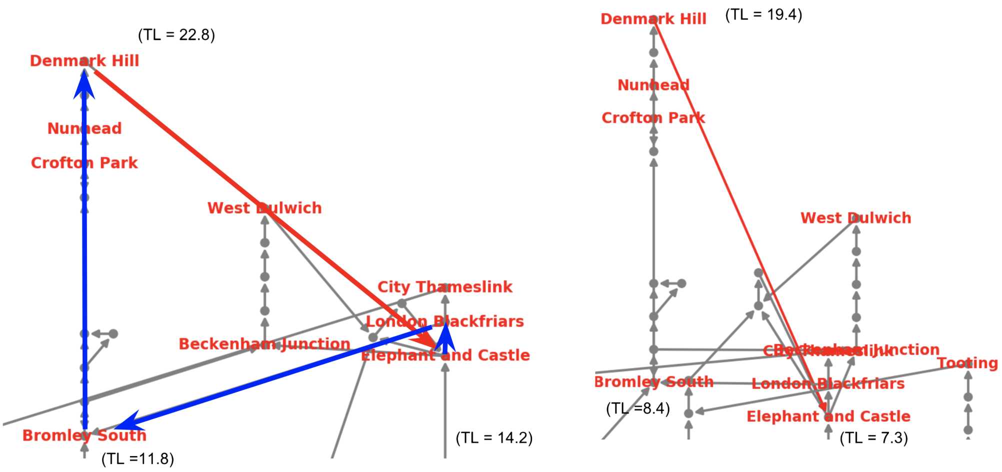

Figure 2. The highlighted loop (figure on the left, before the link removal) is responsible of the elevated trophic incoherence of the network. Removing the link highlighted in red reduces the trophic incoherence and the trophic level of the stations (figure on the right, after the link removal).

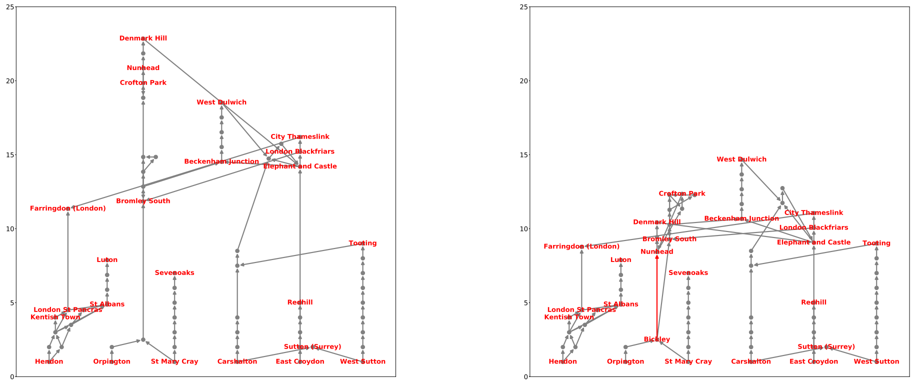

Figure 3. Stations trophic level. On the left the trophic level of the *Thameslink* stations, on the right the trophic level of the same stations when the link from *Bickley* to *Nunhead* is added.

Trophic Coherence Analysis In Figure 3 we show the incoherence gain of the *Thameslink* rail network when a direct train service from *Bickley* to *Nunhead* is added. *Denmark Hill*, the node with the highest trophic level, decreases its level from 22.8 to 11 and *West Bulwich* becomes the node with the new highest trophic level (from 19 to 15). Obviously, adding a link does not remove any loop, but instead it reduces the trophic incoherence adopting data-driven (using morning peak-hour commutation data) changes in the network structure. In particular, this modification suggests a better design for the morning peak hours rides, improving the connection between basal nodes (origin of the commuters) and top nodes (destination of the commuters).
